# Unmanned Aerial Systems and Deep Learning for Safety and Health Activity Monitoring on Construction Sites

**DOI:** 10.3390/s23156690

**Published:** 2023-07-26

**Authors:** Aliu Akinsemoyin, Ibukun Awolusi, Debaditya Chakraborty, Ahmed Jalil Al-Bayati, Abiola Akanmu

**Affiliations:** 1 School of Civil & Environmental Engineering, and Construction Management, The University of Texas at San Antonio, San Antonio, TX 78207, USA; aliu.akinsemoyin@my.utsa.edu (A.A.); debaditya.chakraborty@utsa.edu (D.C.); 2 Department of Civil and Architectural Engineering, Lawrence Technological University, Southfield, MI 48075, USA; aalbayati@ltu.edu; 3 Myers-Lawson School of Construction, Virginia Tech, Blacksburg, VA 24061, USA; abiola@vt.edu

**Keywords:** activity monitoring, construction site, deep learning, safety and health, UAS

## Abstract

Construction is a highly hazardous industry typified by several complex features in dynamic work environments that have the possibility of causing harm or ill health to construction workers. The constant monitoring of workers’ unsafe behaviors and work conditions is considered not only a proactive but also an active method of removing safety and health hazards and preventing potential accidents on construction sites. The integration of sensor technologies and artificial intelligence for computer vision can be used to create a robust management strategy and enhance the analysis of safety and health data needed to generate insights and take action to protect workers on construction sites. This study presents the development and validation of a framework that implements the use of unmanned aerial systems (UASs) and deep learning (DL) for the collection and analysis of safety activity metrics for improving construction safety performance. The developed framework was validated using a pilot case study. Digital images of construction safety activities were collected on active construction sites using a UAS, and the performance of two different object detection deep-learning algorithms/models (Faster R-CNN and YOLOv3) for safety hardhat detection were compared. The dataset included 7041 preprocessed and augmented images with a 75/25 training and testing split. From the case study results, Faster R-CNN showed a higher precision of 93.1% than YOLOv3 (89.8%). The findings of this study show the impact and potential benefits of using UASs and DL in computer vision applications for managing safety and health on construction sites.

## 1. Introduction

The construction industry is one of the most dangerous industries worldwide and the constant exposure of workers to hazardous and complex construction work environments makes them susceptible to various illnesses, injuries, and fatalities [1]. This has remained a serious issue despite efforts by industry associations and regulatory bodies and extensive research to address this problem [2,3]. According to the Occupational Safety and Health Administration (OSHA), about 20% (1061) of worker fatalities in private industry in the year 2019 were in construction, and this accounted for one in five worker deaths for the year [4]. Based on the information from the U.S. Bureau of Labor Statistics, the construction industry has 30% more nonfatal occupational injuries and illnesses than the average industry [4]. The need to reduce nonfatal and fatal injuries is of utmost importance, and an integrated application of cutting-edge sensing technologies and data analysis techniques to tackle this challenge is explored in this conference extension study.

Unsafe behavior and conditions are considered major causes of accidents, and several studies have shown that they account for more than 90% of accidents experienced on construction job sites [1,5]. Thus, the uninterrupted monitoring of workers’ unsafe behaviors and conditions has been recognized as a proactive and active method of mitigating/removing safety and health hazards and preventing accidents on construction sites [6]. Traditional methods for monitoring and analyzing activities on construction sites such as site inspection and observations are expensive, slow, labor-intensive, and prone to inaccurate information due to human error [2,5]. Hence, there is a need for an alternative approach such as the deployment of advanced technologies and techniques like computer vision. Through this method, unsafe behaviors and conditions can be identified and analyzed using a detailed set of information obtained from images and videos containing work conditions and workers’ actions [1,5,7].

Computer vision is a subfield of artificial intelligence (AI) that deals with how computers can obtain a high-level understanding from videos and digital images. Computer vision techniques have proven to be efficient in retrieving relevant data from construction sites such as the rapid and convenient detection and tracking of workers, materials, and equipment. For example, Du et al. [8] proposed a system that can detect hardhats in a video sequence using computer vision techniques. Fang et al. [9] also developed a computer vision-based method to determine if workers are wearing a harness when workers are performing a task at height. Although prior research studies have been conducted on the use of various computer vision techniques for health and safety monitoring and analysis in construction including object detection/tracking and action recognition [5], there are still some drawbacks related to the lack of a fully automatic computer vision-based system. In addition, deep learning (DL) provides a robust opportunity for data analytics that will enable automatic hazard identification [1].

Computer vision research consists of two main steps: data collection and data analysis [10]. Data collection involves the gathering of visual input data such as 2D/3D images, time-lapse images, or videos using sensing devices like stationary cameras, stereo vision cameras, or RGB-D sensors on construction sites. The quality of the collected data is important for effective data analysis. Poor lighting, occlusions, or a cluttered background can decrease the quality of the data collected. As a result, multiple cameras are required to be positioned at strategic places to capture appropriate images. However, it is still impossible to capture the entire construction site using these arrangements, indicating that these modes of data collection have limitations [5,10]. Unmanned aerial systems (UASs) or drones, on the other hand, have been used in several industries for surveillance and monitoring by attaching high-definition cameras and sensors to them [11]. UASs are used in the construction industry for progress monitoring in real time and are also employed by security agencies and the military for search and rescue operations, as well as in the agricultural sector for crop monitoring and weed management [12]. UASs can help overcome the limitations of still cameras.

There has been a recent popularity in the application of UASs for the visual monitoring of construction activities and other processes associated with civil infrastructure systems [13]. According to recent studies, UASs are mainly used in the construction industry for monitoring the progress of work, job site logistics, inspecting construction components for structural integrity, and maintenance assessments [14]. UASs can be very useful for safety managers because they provide numerous advantages, including the ability to move faster than humans, reach dangerous and inaccessible areas of job sites, and be fitted with video cameras, wireless sensors, radar, or communication hardware for the collection and transmission of different types of data in real time [15,16]. In order to provide accurate performance information about the condition of a construction job site, there is a need for versatile UASs with the capacity to collect visual data in form of images and videos (e.g., digital: RGB; thermal: T; depth: D; digital + depth: RGB + D) from the most appropriate locations and viewpoints on the job site [13].

The analysis of the location and behavior of construction workers using job site images has been identified as a means of generating valuable information for safety management and productivity analysis [17]. For the recognition of different object classes, monitoring systems primarily rely on artificial intelligence (AI) through the application of supervised learning. Although several conscious efforts are currently being made to automate the monitoring of construction sites, a thorough understanding of how that system will function as a whole is still challenging [18]. The possibility of automating visual monitoring tasks has increased due to the use of deep learning (DL), otherwise referred to as deep structured learning or hierarchical learning. More specifically, the convolutional neural networks (CNN), a class of DL networks, have been found to be very useful for analyzing visual imagery and surmounting the challenges of the manual observation and recording of hazards on construction sites [1]. This study presents an extension of the initial effort by Awolusi et al. [19] and provides a comprehensive report of the characterization of safety and health hazards for computer vision applications, as well as the development and validation of a framework that integrates UASs and DL for safety and health activity monitoring on construction sites.

In this study, existing literature and reports are first reviewed to characterize the common safety and health hazards on construction sites and the different types of sensors used for imagery data collection such as 2D and 3D data sensing devices. Thereafter, various DL algorithms that can be used to analyze imagery data are reviewed. Based on the reviews, an integrated framework that leverages the use of a UAS and DL is developed for monitoring job site activities, generating critical insights, and disseminating useful information for the improvement of safety performance on construction sites. The framework is then validated using a pilot case study by obtaining imagery data of worker safety and health activities from active construction sites using a UAS and analyzing the data using two different DL algorithms.

The fundamental focus of this research Is to provide an approach for the use of UASs and DL for construction safety and health monitoring. The UASs are used to collect a wide range of data overcoming the limitation of still cameras, while DL is used to analyze the data to proactively monitor safety and health activities and generate useful information for hazard control and accident prevention on construction sites. This study contributes to knowledge in a number of ways. First, the study provides a matrix showing the characterization of common safety and health hazards on construction sites to aid computer vision applications. Second, it provides a validated framework for the effective integration of a UAS and DL for safety, health monitoring, and accident prevention on construction sites. Third, it provides a comparison of the performances of two DL object detection algorithms/models for safety hard hat detection. The findings of the study demonstrate the potential impact of deploying UASs and DL in computer vision applications for safety and health management in construction.

## 2. Literature Review

The construction industry still ranks as one of the riskiest industries in which a high number and rate of occupational illnesses, injuries, and fatalities are recorded annually. A significant amount of research has been conducted in order to improve safety monitoring and eliminate hazards on construction sites. A few of these studies have proposed the use of innovative technology such as UASs, wearable sensing devices (WSDs), the Internet of Things (IoT), and artificial intelligence, among others. Having this array of technologies indicates a need to understand the different hazards and metrics that can be measured, the sensor technologies for data collection, and the AI or DL models/algorithms for data analysis. This section contains a review of the different safety and health hazards on construction sites, UAS applications and sensors that can be used for safety and health monitoring, and computer vision and DL applications in construction.

### 2.1. Safety and Health Hazards on Construction Sites

Construction workers, in general, have a high chance of being hurt on the job site. The construction industry is much different from other industrial sectors due to its complex combination of heterogeneous elements typified by hazardous work environments, materials, equipment, project types, and different categories and levels of fieldworkers and management employees. This level of intricacies makes construction one of the most precarious industries with a disproportionately high rate of injuries, illnesses, and fatalities [20,21]. According to Hamid et al. [20], two major hazards (i.e., physical or safety hazards and health hazards) exist on construction sites.

Physical safety hazards are usually associated with the process of work (manual handling, excavation) or equipment/tools used (scaffolds, power access equipment, ladder, roof work, plant, and machinery) or climatic conditions (snow, rain, high temperatures). Based on the information provided by the Bureau of Labor and Statistics, the Occupation Safety and Health Administration (OSHA) identified four major safety hazards on construction sites called the “OSHA Fatal Four”, which are falls, caught-in or -between, struck by, and electrocution. These hazards according to OSHA account for an overwhelmingly high percentage of construction site injuries and fatalities annually.

A hazard that has a risk of physical injury can cause direct injury to workers on the job site and if severe can cause death. However, a hazard that has a risk of ill health is sometimes only noticed after a long-term period and can cause sickness or death after a certain period. These other categories are referred to as health hazards, which include chemical hazards (solvents, adhesives, paints, toxic dust, etc.), physical hazards (noise, radiation, heat, etc.), biological hazards (infectious diseases), and ergonomic risk factors (heavy lifting, repetitive motions, vibration). The American Industrial Hygiene Association (AIHA) in 2019 also developed a “focus four” for health hazards, which are manual material handling, high noise levels, high temperatures, and air contaminants [22]. According to AIHA, these health hazards present the highest level of health risk to construction workers, and a few studies have identified them as hazards that can cause ill health to workers [20,21]. It is, therefore, important to identify and reduce the risk of these hazards on construction sites.

Monitoring construction activities, including unsafe worker behaviors and unsafe working conditions, is important to proactively eliminate potential hazards and prevent accidents on construction job sites [5]. According to Zhang et al. [23], the occurrence and evolution process of construction safety risk is a random process that results in accidents when the safety threshold is exceeded. Based on the accident root causes tracing model (ARCTM) proposed by Abdelhamid et al. [24], accidents occur due to three root causes (failing to identify unsafe conditions, ignoring identified unsafe conditions, and acting unsafely irrespective of the initial conditions), indicating that common hazards on construction sites are generally caused either by unsafe (worker) behaviors or unsafe (working) conditions.

Unsafe behavior has been defined as unacceptable practices with the potential to contribute to future accidents and injuries [25]. Examples of unsafe behaviors or acts that are commonly observed on construction sites include non-compliance with PPE use, improper lifting, and working or standing too close to heavy construction equipment [26]. Monitoring and changing the behaviors of workers engaging in unsafe acts can help to solve safety problems. On the other hand, an unsafe condition is one in which the physical layout of a workplace or work area, as well as the status of tools, equipment, and materials, violates current safety requirements [24]. Unsafe conditions may be present or develop after the task has commenced and might not be noticed by the worker. Examples of unsafe condition hazards on construction sites include unshored trenches, poor housekeeping, and working in poor physical conditions. Workers cannot always detect unsafe conditions, which is why management relies on safety supervisors to identify and reduce hazards in the processes, equipment, and materials they define. Constant lookout for unsafe conditions and prompt resolution of them can help reduce hazards and prevent accidents on construction sites.

### 2.2. UASs for Safety and Health Monitoring

An unmanned aerial system (UAS), commonly called a drone, is an aircraft or a flying robot that can be remotely controlled by humans or fly autonomously using embedded software-controlled flight plans that work with onboard sensors and a global positioning system (GPS) [27]. The increased use of UASs has brought about major advances in the field of remote sensing technology, resulting in the rapid development and proliferation of unmanned aerial imagery technology [28]. The use of UASs is growing in popularity in the construction industry due to the numerous advantages they offer, such as their ability to access unreachable or dangerous areas, use different types of sensors to collect and provide useful information to users like safety managers to facilitate safety monitoring, and timely interventions on construction sites [29]. In a study conducted by Sebastian et al. [30], UASs were compared to hand-held cameras on the construction site, and it was discovered that UASs were preferable due to their ability to capture various angles and heights, providing detailed coverage of the current status on site. Construction has witnessed different applications of UASs ranging from progress monitoring [13,31] to surveying [31,32], structural inspection [33,34], and safety monitoring [15,35]. Using UASs to capture visual data in some of these applications in construction could lead to remarkable time and cost savings. UASs have a broad range of construction safety applications predominantly in the aspects of construction worker behaviors and site conditions monitoring such as workers’ body positions and the use of appropriate personal protective equipment (PPE), exposed edges or gaps, operations around boom vehicles or cranes, and boom vehicles or cranes close to overhead power lines on the construction site [29].

The features and characteristics of different UASs can vary based on their application and adaptation to the specific tasks they are meant to perform. Since UASs are commonly used for many civilian operations, their classification must consider their different characteristics. UASs are classified as low-altitude platforms (LAP) [36] or high-altitude platforms (HAP) [37] depending on their operating platform. A LAP is a semi-stationary aerial communications platform that operates at less than 10 km altitude [12]. Vertical takeoff and landing (VTOL) vehicles, aircraft, and balloons are the three main types of UASs that fall under this group. A HAP, on the other hand, operates at a very high altitude (above 10 km), and vehicles using this platform can fly at speeds of up to 100 km/h to stay in the upper stratosphere for extended periods [12]. Table 1 summarizes the various types of UASs used in construction, as well as their endurance and altitude ratings.

The most common types of UASs in this HAP category are airships, aircraft, and balloons. Fixed-wing UASs generate lift with their wings, whereas rotary-wing UASs use an engine to propel them forward. Fixed-wing UASs can fly at high speeds for long periods with a simple structure [38]. Although rotary-wing UASs can hover, take off, and land vertically, they have slower speeds and a shorter range of flight than their fixed-wing counterparts. Interest in Quadrotor UASs has also grown due to their low cost and ability to perform vertical takeoff and landing [39]. DJI Phantom, 3DR IRIS, and Parrot AR are the most widely used UAS models [32]. According to Li et al. [40], the design of these different types of UASs impacts energy efficiency, which is a critical performance criterion for their communication and applications.

A few research studies have investigated the use of UASs for safety inspections. To demonstrate their use, all of the studies used videos and images of job sites taken with a UAS, primarily for hazard recognition [34]. Irizarry et al. [15] used a camera-equipped aerial quadcopter that can be piloted remotely with a smartphone to communicate the real-time feed of a job site to the safety managers to perform a usability investigation and a heuristic evaluation. According to the study, real-time imagery, the ability to travel to all areas of the job site, and voice interaction are just a few of the features that will make the drone suitable for use in other construction sectors. Similarly, De Melo et al. [35] investigated a UAS’s ability to capture visual data from a job site to determine compliance with safety regulations using a case study methodology. Cameras are the most common sensors used in UASs for construction applications. For the inspection of construction projects, aerial photographs or videos are first captured using cameras, after which they will be processed or used to create three-dimensional models of the projects. Other types of sensors or sensing technologies that can be used with UASs include LiDAR, Microsoft^®^ Kinect, RFID reader, GPS, noise sensors, thermistors, radar, and infrared. A comparison of common image-sensing devices used with UASs for construction applications is presented in Table 2.

Although the use of UASs for safety planning and monitoring offers several potential benefits, a few drawbacks have also been identified [34]. For instance, different aviation administrations around the world control the flying of UASs in their respective countries, and the regulations they set for such operations must be followed when using UASs for safety-related data collection on construction sites. Wind, rain, and snow have an adverse effect on UASs and will also render UAS operations impossible [34]. Safety and health monitoring using UASs suffer shortcomings associated with their limited energy, short flying time, and processing capabilities [12] but still serve as a great tool for safety managers compared to conventional methods.

### 2.3. Application of Computer Vision and Deep Learning for Safety and Health Monitoring

The computer science fields have witnessed a widespread application of machine learning (ML), a subset of AI operations with applications ranging from speech recognition to natural language processing, robot control, and computer vision [1]. Computer vision (CV) gives computers the ability to record, understand and interpret valuable visual information contained in image and video data and then uses contextual information provided by humans to turn that data into insights used for decision-making [49]. Customary machine learning methods are restricted in their ability to process data in its raw form because designing a function extractor requires a large amount of engineering and domain knowledge [50]. On the other hand, deep learning (DL), which is a subset of machine learning that is built around artificial neural networks (ANNs), relies on computational models influenced by the workings of the human brain [49].

**Figure 1:**
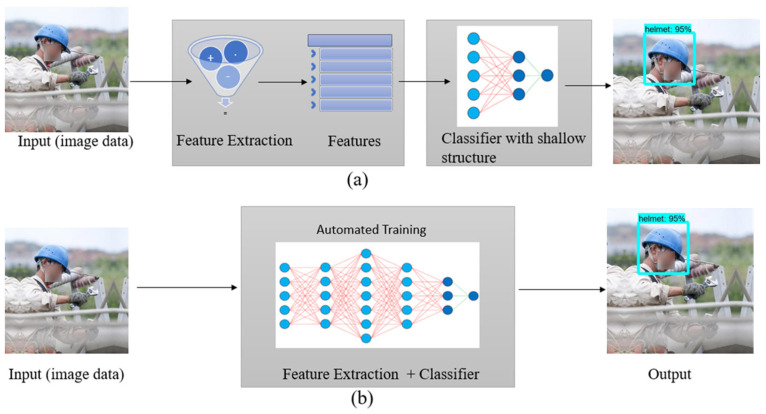
Workflow of (**a**) Traditional Computer Vision and (**b**) Deep Learning.

Unlike traditional CV techniques, DL makes it possible for computer vision engineers to achieve higher accuracy in tasks like image recognition, semantic segmentation, object detection, and simultaneous localization and mapping (SLAM). Although the use of DL has increased recently in different industrial sectors including construction, a fully automatic computer vision-based system for construction safety and health monitoring has not been developed. For instance, while people who are not wearing their PPE can be identified, those who are not complying with this requirement and whether the PPE is being used properly (e.g., the hook of a safety harness not being attached to the rail) still cannot be determined. DL can provide data analytics for the automatic and real-time identification of hazards. As opposed to the traditional CV techniques, which use a pre-programmed neural network, DL applications are trained to leverage the existing large volumes of data that can be generated, requiring less expert analysis and fine-tuning. Unlike the traditional CV algorithms, which are more domain-specific, DL algorithms are more adaptable and versatile because DL models such as the convolutional neural network (CNN) models and frameworks can be re-trained using a custom dataset for any use case [49,51,52]. Figure 1 illustrates the difference between the traditional CV workflow and DL workflow as an example of an object (i.e., PPE) detection application for safety compliance on construction sites.

Other DL models that can be used for computer vision are the “Boltzmann family”, which includes deep belief networks (DBNs), deep Boltzmann machines (DBMs), and stacked (denoising) autoencoders (SdAs) [53,54]. DBNs and DBMs are DL models that use the restricted Boltzmann machine (RBM) as a learning module and thus belong to the “Boltzmann family”. The restricted Boltzmann machine (RBM) is a generative stochastic neural network. DBNs have guided connections to the lower layers and undirected connections at the top two layers, forming an RBM. Both layers of the network have undirected connections in DBMs [54,55,56]. In the same way that DBNs use RBMs as a part, stacked autoencoders use the autoencoder as their main building block [54]. Image recognition, object detection, behavior and movement recognition, face recognition, human pose prediction, image retrieval, and semantic segmentation are only a few of the visual comprehension tasks that these DL models have been used for [54,56,57,58]. Feature learning is a peculiar capability of CNNs; it enables them to automatically learn features from a dataset. CNNs are also transformation invariant, which is useful in computer vision. In contrast to DBNs/DBMs and SdAs, which can run unsupervised, CNNs rely heavily on the presence of labeled data. Both CNNs and DBNs/DBMs are computationally intensive when it comes to training, while SdAs can be trained in real-time under some conditions [54].

### 2.4. Convolutional Neural Networks in Computer Vision

In recent years, the growth of CNNs has had a huge impact on the field of computer vision, as it has been able to outperform other deep neural networks in areas like image recognition, object detection, and segmentation [1,49]. Convolutional layers, pooling layers, and connected layers are the three major types of neural layers in a CNN and each layer has a distinct function [54]. Learning time can be sped up by using convolutional layers. A CNN convolves the entire picture as well as the intermediate feature maps, resulting in a variety of feature maps. It has been proposed as a replacement for completely linked layers in many works [54]. Pooling layers are responsible for reducing the input volume’s spatial dimensions (width and height) in preparation for the next convolutional layer. The most widely used techniques are average pooling and full pooling. Since the reduction in size results in a simultaneous loss of information, the process performed by this layer is also known as subsampling or downsampling. The depth dimension of the volume is unaffected by the pooling layer. It is advantageous to the network because it prevents overfitting [59]. Fully connected layers in the neural network are used to perform high-level reasoning. All activation in the previous layer is completely connected to neurons in a fully connected layer. The 2D feature maps are gradually transformed into a 1D feature vector by completely connected layers. The resulting vector could be fed into a variety of classification categories. Two deep convolutional networks (Faster R-CNN and YOLOv3) commonly used for object detection are described as follows.

#### 2.4.1. Faster R-CNN

Faster R-CNN introduces a region proposal network (RPN) that can generate high-quality region proposals and be used to detect and classify objects based on the region proposals and share full-image convolutional features with the RPN. It is an object detection method proposed by [60] in 2015. On a graphics processing unit (GPU), the Faster R-CNN has a frame rate of 5 frames per second, which makes it a viable object detection system in terms of both speed and precision.

The Faster R-CNN approach consists of three phases. First, the characteristics of a photograph are extracted, and then to create a convolutional feature map, the network uses the CNN method to process the whole image with many convolutional layers and max-pooling layers [9]. The second module is a deep, fully convolutional network that proposes regions based on the features [60]. Judging whether a person is wearing a hardhat only based on the feature maps is challenging because the image usually contains numerous superfluous things and persons always appear very little in a whole image. As a result, the foreground regions, which may include humans, must be distinguished from background regions before excluding the latter. For recognizing non-hardhat-use (NHU), only foreground regions are used. The third module uses proposed regions and extracted fractures to determine if the proposed region is a worker wearing a hardhat or not [60].

Faster R-CNN has three major advantages over other methods used for hardhat detection in previous studies: (i) it is robust in dealing with different complexity of construction site environments; (ii) it can fulfill the need for a practical engineering approach due to its high precision; and (iii) real-time monitoring and detection of hard hats can be achieved due to its short processing time [9,61,62].

#### 2.4.2. YOLOv3

The You Only Look Once (YOLO) algorithm is a cutting-edge real-time object detection system that uses CNN principles [63,64]. YOLO divides the input image into a S × S grid [64,65]. Each grid cell predicts only one object and a fixed number of boundary boxes. It predicts boundary boxes (B), including position information of the bounding box for each grid cell, with one box confidence score for each box [61]. Then, regardless of the number of boxes B, it detects only one object. Finally, it forecasts conditional class (C) probabilities (one per class for the likeliness of the object class). Each boundary box is made up of five elements: (center point coordinates x, y, width w, height h) and a box confidence score [63,66]. The confidence score reflects the box’s likelihood of containing an object and the accuracy of the boundary box.

YOLOv3 improved on YOLO by adding batch normalization, high-resolution classifiers, convolutions with anchor boxes, multi-scale training, and joint classification and detection. YOLOv3 is inspired by Faster R-anchor CNN’s box. To obtain good priors, it abandons the manually selected anchor box and performs k-means clustering on the dimensions of bounding boxes [67]. This method is used by YOLOv3 to obtain 9 cluster centers, which can better cover the characteristics of the train set’s ground truth. YOLOv3 has acquired image features at different scales and greatly improved the detection of small targets as a result of predictions on multiple-scale feature maps [27]. YOLOv3 assigns several anchor boxes to each scale feature map based on the length and width of the anchor boxes, combining the anchor box and multi-scale prediction idea. YOLOv3 uses the Darknet53 network as the backbone. YOLOv3 is an advanced CNN architecture with a mean AP (Accuracy precision) metric that is comparable to other similar architectures such as Faster R-CNN, SSD513, RetinaNet, and DSSD [68] but with a faster prediction rate. Luo et al. [69] found that YOLOv3 is effective at detecting multiple workers on construction sites.

## 3. Materials and Methods

**Figure 2:**
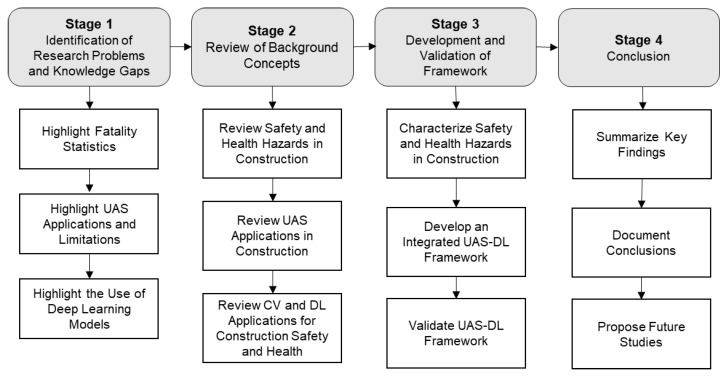
Research Process.

This section describes the research method implemented for this study. This study was conducted using a combination of tasks as depicted in Figure 2 below. The entire process illustrated in Figure 2 can be summarized into four stages which include (i) identifying research problems and knowledge gaps; (ii) reviewing background concepts; (iii) developing and validating the framework; and (iv) documenting the conclusion of the study. The study began with a review of fatality statistics in the construction industry and the current methods of safety and health activity monitoring on construction sites to identify the research gaps and needs. Thereafter, UAS applications and their limitations along with the use of DL models were reviewed to identify their existing and potential applications for construction safety and health monitoring. This was followed in stage 2 by a detailed review of the concepts highlighted in state 1.

Based on the reviews conducted in stage 2, the safety and health hazards on construction sites were characterized in stage 3 by determining and mapping the different attributes of the hazards (including their source categories, metrics that can be measured, etc.) with computer vision applications comprising of data capturing sensors, data types/features, and deep learning models to be used for analysis. Following that, an integrated framework for the monitoring of safety and health hazards of construction sites using UAS and DL was developed, and a case study of hardhat detection on construction sites was implemented to test the framework. The results of the case study were analyzed, and the findings were discussed and compared to those obtained from the existing studies, after which conclusions were made in the final stage with accompanying recommendations for future studies.

### 3.1. Construction Safety and Health Hazards Characterization for Computer Vision Applications

**Figure 3:**
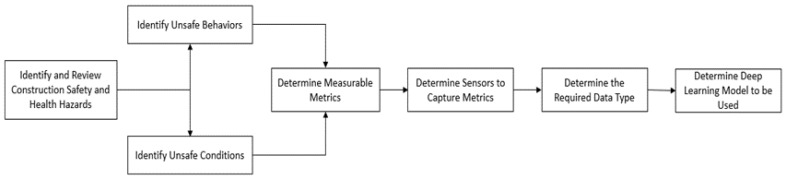
Characterizing construction safety and health hazards for CV applications.

For the characterization, incident statistics reports, OSHA publications, and existing research studies on construction safety and health hazards were reviewed to identify the common hazards and their attributes, such as their source categories (unsafe behavior or conditions), metrics to be measured, etc. The different UAS sensors used to collect or capture the data type of each hazard based on their metrics were identified. Thereafter, the types of deep learning techniques (such as RNN, CNN for object detection and action recognition, etc.) used to analyze the captured data in terms of detecting and measuring the appropriate metrics were identified. These different components were mapped based on existing applications of computing vision as reported in published studies. The steps adopted for the characterization are depicted in Figure 3.

### 3.2. Development of the Framework

Based on the reviews conducted and the characterization of construction safety and health hazards for computer vision applications, an integrated framework that leverages the use of UAS and DL for the continuous monitoring of safety and health activity for worker safety performance improvement on construction sites was developed. It is expected that this framework will be used to automate data collection (using UASs) and analysis (using DL) to identify unsafe worker behaviors and site conditions in order to provide real-time feedback for accident prevention on construction sites.

### 3.3. Pilot Case Study to Implement the Framework

A pilot case study was conducted to implement the integrated UAS-DL framework. A DJI Mavis 2 Pro UAS (with 1 CMOS, 20 megapixels cameras; 4K UHD and 10-bit D-log video; 31 min flight time, and; 907 g weight) was flown over different active construction sites in Texas, United States to capture quality images and videos of workers engaging in different work processes and activities such as foundation work, scaffolding, trenching, etc. The images and videos were collected over several months to capture a sizable amount of data that reflects different construction tasks/activities at different phases. The case study scenario used was safety hardhat (helmet) detection.

The study employed the Faster R-CNN and YOLOv3 architecture using the open-source TensorFlow framework and Keras backend. Keras is a high-level network API written in Python that uses the TensorFlow framework for implementation. Google Colab, a web-based notebook that allows writing and execution of arbitrary Python code through the browser, was used to execute the codes, and NVIDIA T4 Tensor Core GPU available on Google colab was used for the model training. The primary environment consists of Python3, Tensorflow, pip, OpenCV, and Keras, which was run on Google Colab and operated from an Intel Core i7-8565U 1.8 GHz CPU and Intel HD Graphics GPU with a 16 GB RAM hardware. To reduce training time and save computer resources, the generated dataset combined the COCO dataset with the pre-trained faster_rcnn_resnet152_v1_640×640_coco17 for one of the models and pre-trained YOLOv3 for the other model to learn the safety helmet’s features.

**Figure 4:**
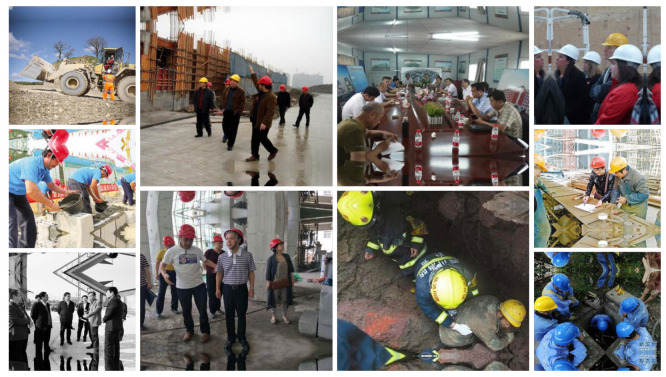
Image dataset from Roboflow.

**Figure 5:**
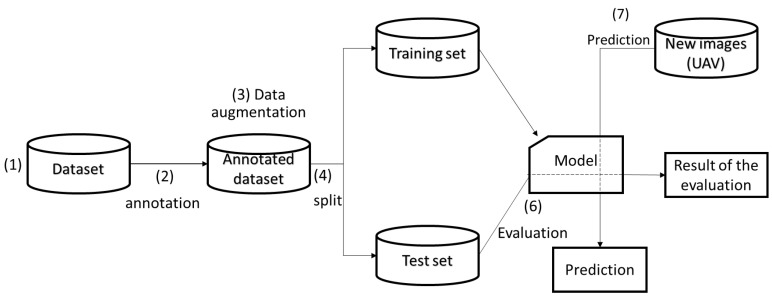
Model flowchart.

**Figure 6:**
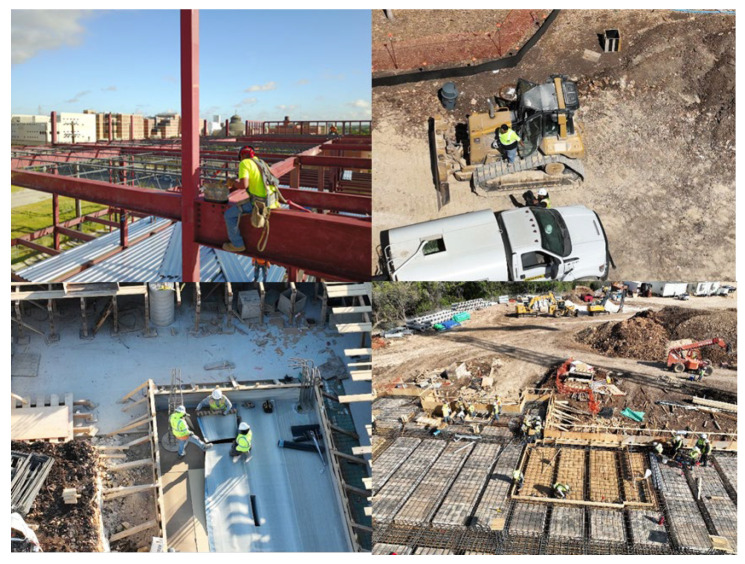
Images captured using UAS.

An off-the-shelf dataset available on the Roboflow website (https://public.roboflow.com/object-detection/hard-hat-workers/1, accessed on 17 April 2021) with sample images shown in Figure 4 was used to train the model. The dataset consisted of 7041 preprocessed and augmented images with a 75/25 training and testing split. The training set was used to train the model to determine the parameter of the model, while the testing set was used to evaluate the generalization ability of the final model [70]. The number of classes was set to 2, “Helmet” and “Head”. Figure 5 shows the flow chart used for developing the models. The images captured (using DJI Mavis 2 Pro by DJI, Shenzhen, China) from the active construction sites (Figure 6) were also used to validate the model. Due to the limited computational power of the machine used, the dataset images were resized several times in order to train the model. 

#### 3.3.1. Training Using Faster R-CNN

The training was done using TensorFlow 2.0 on Google Colab. During the training, the TensorFlow board was used to capture the overall loss values. After the model had been trained, it was validated with images captured by the UAS and some construction images from the dataset. To test the model, 1766 images were inputted into it, and the images detected were output. Confidence score predicted labels, and bounding boxes surrounding the predicted part in the images were shown in the images.

#### 3.3.2. Training Using YOLOv3

The model for training was made by combining the configuration (cfg) file and weights file of YOLOv3. An h5 model file was generated, which had 252 layers. The model layers include 23 adding layers, 72 batch normalization layers, 75 2D convolutional layers, 72 leaky rectified linear unit (ReLu) layers, five 2D zero-padding layers, two concatenate and up-sampling layers, and a YOLO loss layer. The data after all epochs before unfreezing all layers and three epochs after unfreezing all layers in training was stored as a new model. For better training, reducing the learning rate of a change in validation loss was not less than 0.1. To avoid overfitting, early stopping of a change in validation loss is nil for the last 1000 epochs used.

#### 3.3.3. Metrics for Performance

Precision and recall are two metrics often used to assess the trained model’s reliability and performance. The ratio of true positive (TP) to true positive and false positive (TP + FP) is known as precision. The number of helmets detected is TP + FP. The ratio of true positive (TP) to true positive and false negative (TP + FN) is known as recall. The exact number of helmets is represented by TP + FN.

(1) $$\mathrm{Precision}=\frac{\mathrm{TP}}{\mathrm{TP}+\mathrm{TF}}$$

(2) $$\mathrm{Recall}=\frac{\mathrm{TP}}{\mathrm{TP}+\mathrm{FN}}$$

## 4. Results and Discussion

This section presents the results of the study, their analysis, and discussion of findings. First, a matrix containing the characterization of safety and health hazards for computer vision applications in construction is presented. This is followed by the presentation of the UAS-DL framework developed and the validation of the framework using a pilot case study.

### 4.1. Matrix of Construction Hazards Characterization for Computer Vision Applications

Table 3, first presented in Awolusi et al. [19], shows a matrix of the characterization of the common safety and health hazards for computer vision applications in construction. This matrix shows the analysis (or breakdown) of common hazards and their attributes including source categories (i.e., unsafe behavior or conditions), measurable metrics, the different UAS sensors for data capture/collection, the data type, and the DL techniques that can be used to analyze the captured data. For instance, the potential for a struck-by hazard, when a pedestrian worker is close to a piece of heavy equipment (which could be an unsafe behavior or condition), can be averted when a UAS (fitted with HD camera and GPS sensors) detects and captures the location and proximity of the equipment and worker. The UAS sensor then provides the data in the form of 2D or 3D images or videos, which are then analyzed by a CNN through object detection and object tracking to provide useful information and feedback in real time for accident prevention. Kim et al. [71] evaluated this concept of UAS application in their investigation and obtained a promising level of accuracy. Similarly, a health hazard like improper lifting causing an awkward worker body posture or orientation can be detected by a UAS with an HD camera sensor which produces 2D or 3D images or videos processed using CNN for object detection and action recognition.

### 4.2. Framework for Safety and Health Activity Monitoring Using UASs and DL

**Figure 7:**
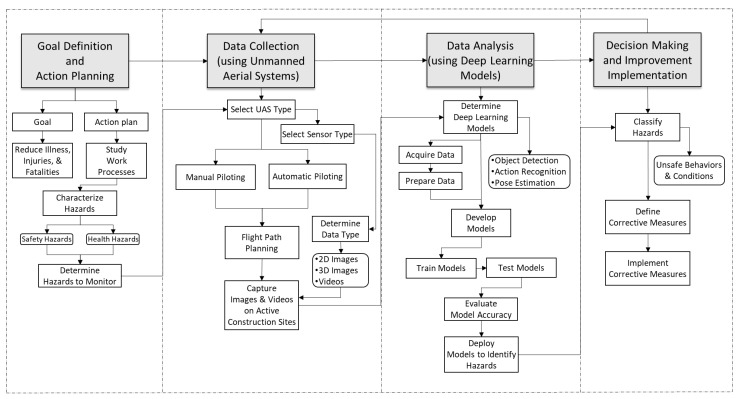
Construction Safety Activity Analysis Framework Using UASs and DL.

This integrated framework utilizes UASs and DL for the continuous monitoring of safety and health activity to improve worker safety performance on construction sites. It is expected to be deployed to automate the collection of safety and health activity data (using UASs) and analysis the analysis of the data (using DL) to detect hazards and identify unsafe worker behaviors and site conditions for the provision of real-time feedback for accident prevention on construction sites. The framework has four major phases illustrated in Figure 7 and described as follows.

#### 4.2.1. Phase 1: Goal Definition and Action Plan

In this phase, the safety and health performance goals and objectives, along with the categories of measurement and metrics, are defined by the safety manager and other stakeholders in the organization. Thereafter, an action plan is initiated, first by studying the work processes on the construction project to identify the different possible hazards associated with different operations and tasks. The hazards are then characterized into different categories, such as safety hazards and health hazards, unsafe behaviors, and unsafe conditions, and the respective measurable metrics. The procedure will be championed by a team made up of the organization’s safety personnel. The different types of hazards on the construction project such as workers not wearing hard hats, workers standing too close to trenches, inappropriate lifting, and standing on the haul road near heavy equipment are among the activities and hazards that must be identified by all stakeholders of the organization.

#### 4.2.2. Phase 2: Data Collection (Using a UAS)

In this phase, the construction site activities are monitored using a UAS equipped with appropriate sensors to identify critical hazards including unsafe behaviors and conditions. A flight protocol such as the one proposed by Kim et al. [18] consisting of three steps (pre-flight step, flight step, and post-flight step) can be used to plan the flight path. Flight mission plans, including targeted sites (points of interest) and takeoff and landing locations, are determined based on the pre-flight discussion. The flight team determines the UAS to be used and examines the UAS conditions before flights once the flight parameters and pertinent information have been identified. The flight operator will communicate with observers, project engineers, and safety supervisors while the UAS collects or captures visual data. More than one flight might be required during this phase depending on the need impacted by site variables such as the size of the project, the number and nature of activities taking place, etc. After all the flights have been completed, the acquired images and videos will be processed and transmitted to external storage. The UAS can be flown manually or preprogrammed with a flight path with scheduled flying times.

#### 4.2.3. Phase 3: Data Analysis (Using DL)

This is the phase at which the developed DL model is deployed to analyze the collected data for hazard detection. The developed DL model could be object detection, action recognition, pose estimation, or a combination of these and other computer vision tasks depending on the types of hazards on the construction site. Depending on the type of model to be trained, the data needed to train the model are acquired from appropriate sources. To achieve homogeneity, the collected data are cleaned and preprocessed. After the model is trained, tested, and its accuracy, speed, and efficiency are assessed, the model is deployed for the identification and analysis of hazards on the construction site.

#### 4.2.4. Phase 4: Decision Making and Improvement Implementation

This is the final stage at which decisions are made by the management of the organization for improvements based on the results of the data analysis. The report generated from the monitoring process (i.e., data collection and analysis) will include hazards that should be mitigated or eliminated and unsafe behaviors and conditions that should be corrected, together with recommendations for immediate intervention and modification. Appropriate plans are made by the safety personnel to implement the corrective measures by providing real-time alerts to workers and training to eliminate unsafe behaviors and correct unsafe working conditions on the construction site.

### 4.3. Training and Testing Results

The results of the model training using Faster R-CNN and YOLOv3 are presented as follows. The two models were trained using the same datasets.

#### 4.3.1. Faster R-CNN

**Figure 8:**
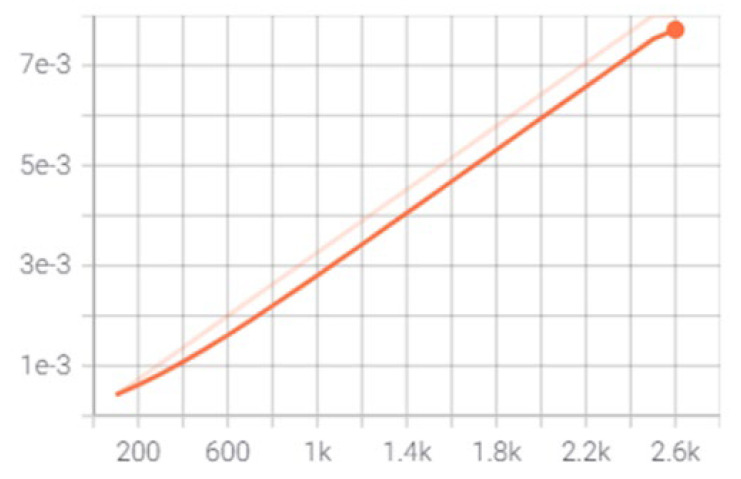
Learning rate.

**Figure 9:**
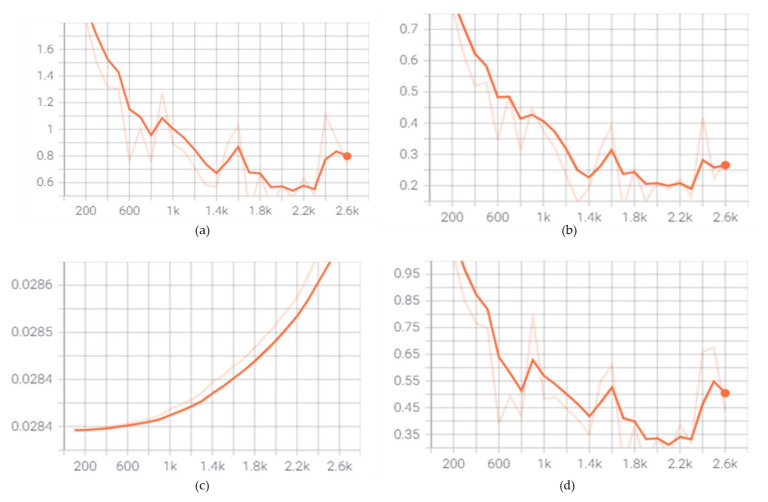
(**a**) Total loss (**b**) localization loss (**c**) regularization loss (**d**) classification loss.

Figure 8 illustrates the pace at which the algorithm updates and learns the values of the parameter estimates. The tensor board was used to capture the loss functions during the training process. Figure 9a shows that the total loss values fall gradually at the beginning of the training and gradually converge towards the end. The disparities between the true and predicted values are the loss function values. The model’s training process is represented by changes in the loss function. The lower the values, the better the model has been trained. The loss function has an impact on the training process because it signals when it is finished. Figure 9a–c demonstrate the differences in the local loss, regularization loss, and classification loss. The classification and localization loss values decline dramatically at the start of the training and then gradually as the training progresses until about 1500 steps. The convergence of the loss function shows that the training has been completed.

**Figure 10:**
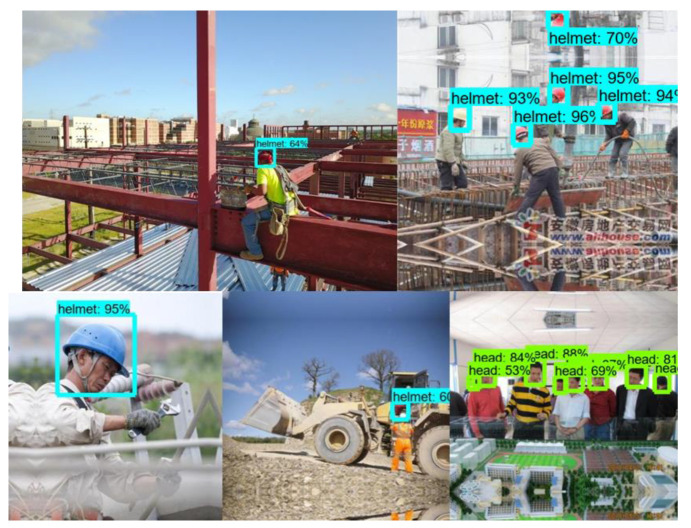
Testing results (Faster R-CNN model).

**Figure 11:**
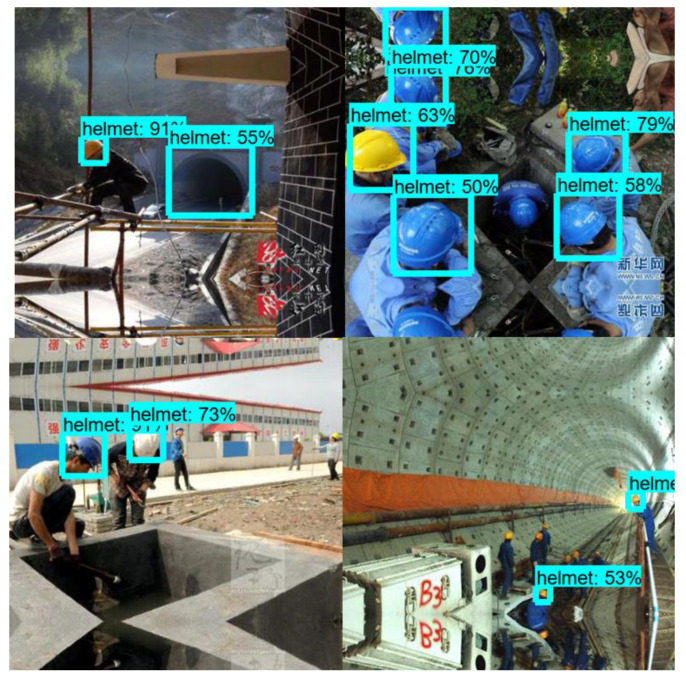
Testing results shown some detection errors (Faster R-CNN model).

The trained model had a precision of 95% and a recall of 80%, indicating that the model performs well in detecting safety helmets. The probability of detecting safety helmets is depicted in Figure 10 and Figure 11 below.

However, several inaccuracies were found in the output images (Figure 11), indicating that the detection model had some flaws. The safety helmets were detected in wide photographs and close-up images; however, there were some false detections in images with low light intensity and images with shapes similar to those of a safety helmet. The helmets were not detected in several of the photographs when only a little section of the helmet was seen.

#### 4.3.2. YOLOv3

**Figure 12:**
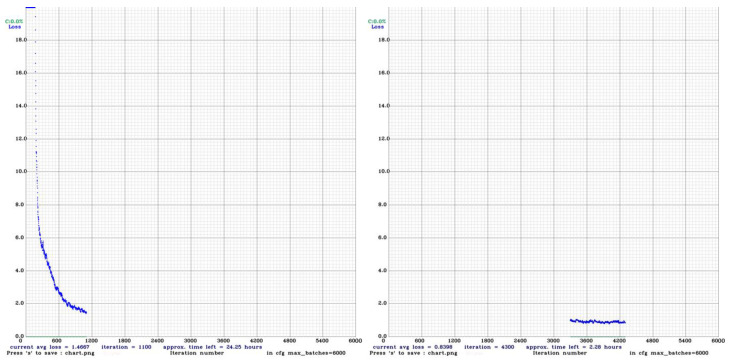
Training loss vs. Iteration.

A batch size of 4 was used for the training. The Adam optimizer, with an initial learning rate of 0.001, was used for training. An early stoppage was used, with the first training stopped after 1100 iterations, and the second one after 4200 iterations as shown in Figure 12 below. The training progress of the network was monitored using loss and accuracy.

**Figure 13:**
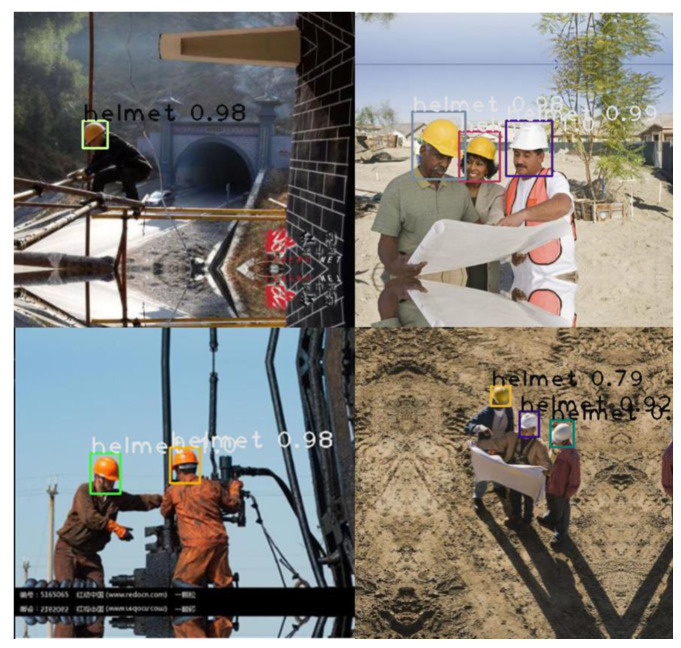
Testing results (YOLOv3 model).

**Figure 14:**
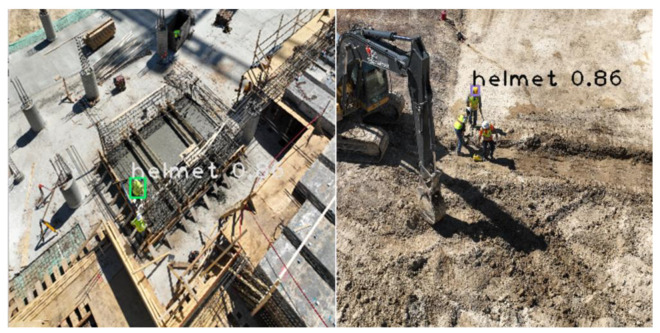
Testing results on UAS images (YOLOv3 model).

Some detection errors were also found in the output images, indicating that the detection model had some flaws. The safety helmets could be detected in wide photographs and close-up images (Figure 13); however, there were some false detections in images with low light intensity and UAS images taken from a far distance as shown in Figure 14. The helmets were not detected in several of the photographs when only a little section of the helmet was seen and where people were small due to the distance at which the images were taken. These errors are major because images taken using UASs were not included in the training data set. Images taken using UASs were taken from different angles and had different resolutions compared to images taken using still cameras. The objects sometimes appeared very small. UAS images need to be included in future model training for better detection.

#### 4.3.3. Summary of Performance

A confusion matrix was used to understand the performances of the trained models, in which human interpretations and model predictions were counted and marked on a table. The precisions of the models’ performances were assessed. For this, true positive (TP), true negative (TN), false positive (FP), and false negative (FN) values of each class were counted and tabulated. Faster R-CNN showed a higher accuracy than YOLOv3 as seen in Table 4 below. This result is in line with a previous study by Fang et al. [9], who found that Faster R-CNN had a higher precision compared to the other model tested in their study.

## 5. Conclusions

The proactive and active monitoring of hazards in high-risk work environments like construction sites is important to ensure the safety of all workers. Leveraging technologies such as UASs and DL for proactive and active safety and health monitoring and analysis can be very effective in reducing the prevalence of fatal and non-fatal injuries on construction sites. Objective and accurate data collection and analysis can be accomplished with this class of technologies to give real-time feedback for enhancing safety and health management on construction sites. In this study, a review of the safety and health hazards that construction workers are exposed to was conducted. They were characterized and mapped to the metrics that can be measured and the DL models that can be used to analyze them. Thereafter, a framework for construction safety and health activity analysis and monitoring using UASs and DL was developed, and a pilot case study was conducted to validate the framework.

This study advances knowledge in a variety of ways. First, the findings of this study provide useful information through which UASs and DL can be effectively deployed to monitor worker safety and health activities for accident prevention on construction sites. The matrix of characterized safety and health hazards provided in this study is expected to facilitate computer vision applications and can be adapted and applied to various types or scales of construction work processes. The framework developed provides an approach for safety and health monitoring and accident prevention on construction sites. The study also presents a comparison of the performances of two DL object detection algorithms/models for safety hard hat detection. The findings of this investigation show the potential benefits of using UASs and DL in computer vision applications for managing safety and health in the construction industry.

In terms of the limitation of the study, adding more UAS-captured data (i.e., images) to the training dataset could have further increased the precision of the detection. Further improvements or modifications to the models used or exploring other algorithms could increase the precision of the detection of images taken from high altitudes and images where the detection targets were very small. The pilot case study conducted is also limited in that only one safety issue scenario was used for the validation. Also, the validation did not cover real-time UAS monitoring for decision-making and implementation of improvements, which should be the subject of future studies following this investigation.

Future studies should explore the use of data containing more UAS-captured images for model training. The whole framework should also be implemented on an active project so that all the components can be validated to determine the framework’s effectiveness and identify areas of improvement. Additional DL models can be developed and tested as part of the framework, and the integration of the framework with the Internet of Things (IoT) for real-time data collection, analysis, and improvement implementation should be explored.

## Figures and Tables

**Table 1:** Common UASs Used in Construction.

UAS Type	Endurance/Altitude	Applicable Environment
Quadrotor Helicopter	Less than 1 h/LAP	Indoor/Outdoor
Fixed Wing Aircraft, UAS	Up to 50 min/up to 1640 ft	Outdoor
Quadrotor, UAS	Up to 25 min/up to 656 ft	Indoor/Outdoor
Multi Rotor UAS	20 min/LAP	Indoor/Outdoor
Parrot AR.Drone	12 min/164 ft	Indoor/Outdoor
MikroKopter L4-ME Quadcopter	Up to 20 min/up to 810 ft	Indoor/Outdoor

**Table 2:** Comparison of Image Sensing Devices.

Device	Data Type	Sensing Range	UAS Mountable	Sample Use Case
Stationary camera	2D images	Long	No	Worker detection [41]
360 cameras	2D images	Long	Yes	Bridge inspection [42]
Portable camera	2D images	Long	No	Object identification [43]
Flash LADAR	3D images	Short (<10 m)	No	Resource detection and tracking [44]
Stereo vision camera	2D and 3D images	Long (more accurate <10 m)	Yes	Structural health monitoring [45]
RGB-D sensors	3D images	Short (<5 m)	Yes	Crack identification [46]
Thermal inferred cameras	2D images	Long	Yes	Building envelope inspection [47]
3D laser scanner	3D images	Long	Yes	Infrastructure inspection [48]

**Table 3:** Characterization of Common Construction Safety and Health Hazards [19].

	Hazards	Unsafe Behavior	Unsafe Condition	Metrics	UAS Sensors	Data Type	DL Techniques	Source
Safety Hazards	Falls from height	✓	✓	Body posture	HD camera	2D images, videos	CNN—Object detection	[9,72]
	Caught-in or -between	✓	✓	Proximity detection	HD camera	2D images, videos	CNN—Object detection	[71]
	Struck-by object	✓	✓	Proximity detection, Location tracking	HD camera, GPS	2D images, 3D images, videos	CNN—Object detection and object tracking	[71]
	Electrocution	✓	✓	Proximity detection, location tracking	HD cameras, GPS	2D images, 3D images, videos	CNN—Object detection and object tracking	[71]
	Slips and trips	✓	✓	Body posture, body speed	HD camera	2D images, 3D images, videos	CNN—Object detection and action recognition	[17,73]
	Cave in	✓	✓	Location tracking	HD camera, GPS	2D images, 3D images, videos	CNN—Object detection and object tracking	[6]
	Not using PPE	✓		Object detection	HD camera	2D images videos	CNN—Object detection	[9,61]
Health Hazards								
	Loud Noise		✓	Noise level	Acoustic sensor	Audio recording	RNN—LSTM	[12,22,73]
	High temperatures		✓	Body temperature	Thermal cameras	2D images, thermal images, videos	CNN—Object detection	[12,22]
	Fire and explosion		✓	Smoke and fire detection	Thermal cameras	Thermal images, videos		[12,22]
	Improper lifting	✓		Body posture and orientation	HD camera	2D images, 3D images, videos	CNN—Object detection and action recognition	[17,22]

CNN—Convolution Neural Network, RNN—Recurrent Neural Network, LSTM—Long Short-Term Memory.

**Table 4:** Classification Report for Test Dataset.

	TP		FP		FN		Precision (%)		Recall (%)	
	Faster R-CNN	YOLO v3	Faster R-CNN	YOLO v3	Faster R-CNN	YOLO v3	Faster R-CNN	YOLO v3	Faster R-CNN	YOLO v3
Helmet	3478	3201	254	371	381	386	93.1	89.8	90.1	89.2
Head	1563	1358	158	161	181	169	90.1	89.4	89.6	88.9

## Data Availability

The data presented in this study are available upon reasonable request from the corresponding author. The data are not publicly available due to privacy restrictions.
